# 驱动基因阳性且PD-L1高表达的非小细胞肺癌患者病理特征及靶向治疗疗效评估的真实世界研究

**DOI:** 10.3779/j.issn.1009-3419.2021.104.02

**Published:** 2021-02-20

**Authors:** 卉 张, 新杰 杨, 琨 李, 敬慧 王, 嘉林 吕, 曦 李, 新勇 张, 娜 秦, 权 张, 羽华 吴, 丽 马, 飞 盖, 瑛 胡, 树才 张

**Affiliations:** 1 101149 北京，首都医科大学附属北京胸科医院/北京市结核病胸部肿瘤研究所肿瘤内科 Department of Medical Oncology, Beijing Chest Hospital, Capital Medical University/Beijing Tuberculosis and Thoracic Tumor Research Institute, Beijing 101149, China; 2 101149 北京，首都医科大学附属北京胸科医院/北京市结核病胸部肿瘤研究所病理科 Department of Pathology, Beijing Chest Hospital, Capital Medical University/Beijing Tuberculosis and Thoracic Tumor Research Institute, Beijing 101149, China; 3 361000 厦门，厦门艾德生物医药科技股份有限公司 Amoy Diagnostics Co., Ltd, Xiamen 361000, China

**Keywords:** 肺肿瘤, 驱动基因, 程序性死亡配体1, 靶向治疗, Lung neoplasms, Driver mutation, Programmed death-ligand 1, Targeted treatment

## Abstract

**背景与目的:**

驱动基因突变阳性患者行靶向治疗，驱动基因阴性但程序性死亡配体1（programmed death-ligand 1, PD-L1）高表达患者行免疫抑制剂治疗，是晚期非小细胞肺癌（non-small cell lung cancer, NSCLC）患者一线治疗的首选，但对于驱动基因阳性且PD-L1高表达患者的治疗选择仍值得探究。

**方法:**

以315例NSCLC患者为研究对象，分析驱动基因阳性且PD-L1高表达患者的临床病理特征及靶向治疗疗效。

**结果:**

本研究纳入的315例NSCLC患者中，驱动基因突变总阳性率为62.2%，PD-L1高表达率（≥50.0%）为11.2%，驱动基因阳性且PD-L1高表达的患者比例为10.7%。其中表皮生长因子受体（epidermal growth factor receptor, *EGFR*）突变、*KRAS* 突变、*ALK*融合、*BRAF*突变和*MET* 14外显子跳跃突变患者中均有PD-L1高表达，比例分别为7.8%（11/141）、18.2%（4/22）、23.1%（3/13）、50.0%（2/4）和100.0%（1/1）。*EGFR*突变且PD-L1高表达患者主要为Ⅳ期肺腺癌患者，*KRAS*突变且PD-L1高表达患者主要为有吸烟史的患者。其中详细跟踪了两例分别为*ALK*融合阳性且PD-L1高表达（90.0%）和*EGFR* L858R突变且PD-L1高表达（70.0%）患者的靶向治疗全过程，两例患者总生存期分别仅为5个月和2个月。

**结论:**

NSCLC患者各驱动基因突变与PD-L1高表达共存的比例和临床病理特征有较大差异。发生敏感突变且PD-L1高表达的患者靶向治疗疗效和预后可能更差。

## Abstract (en)

**Background and objective:**

Targeted therapy for patients with driver genes positive and immunotherapy for patients with driver gene-negative but high programmed death ligand 1 (PD-L1) expression are the standards of first-line treatment for patients with advanced non-small cell lung cancer (NSCLC). The treatment options for patients with driver gene positive and high PD-L1 expression are still worth exploring.

**Methods:**

The characteristics of 315 patients with NSCLC were identified to analyze the clinicopathological characteristics of patients with driver gene positive and high PD-L1 expression, and the efficacy of targeted therapy.

**Results:**

Among the 315 patients, the total positive rate of driver genes was 62.2%, and the high PD-L1 expression rate (≥50.0%) was 11.2%. The proportion of patients with driver gene positive and high PD-L1 expression was 10.7%. PD-L1 was highly expressed in patients with epidermal growth factor receptor (*EGFR*) mutation, *KRAS* mutation, *ALK* fusion, *BRAF* mutation, and *MET* 14 exon skip mutation, the proportions were 7.8% (11/141), 18.2% (4/22), and 23.1%, (3/13), 50.0% (2/4) and 100.0% (1/1) respectively. *EGFR* mutation positive with PD-L1 high expression was mainly in patients with stage IV lung adenocarcinoma. *KRAS* mutation positive with PD-L1 high expression was mainly in patients with a history of smoking. Among them, two patients were followed in detail for targeted therapy, who with *ALK* fusion-positive and PD-L1 high expression (90.0%), *EGFR* L858R mutation and PD-L1 high expression (70.0%) respectively. The total OS of the patients was 5 months, 2 months.

**Conclusion:**

The high PD-L1 expression rate in NSCLC patients with different driver gene mutations was variable, which maybe correlated with distinct clinicopathological characteristics. Patients with sensitive mutations and high PD-L1 expression may be less benefit from targeted therapy and have poor prognosis.

我国肺癌每年新发病例约733,300例，死亡病例约610,200例，发病率和死亡率均居所有肿瘤的第一位^[1]^。非小细胞肺癌（non-small cell lung cancer, NSCLC）是肺癌中最常见的类型，占所有肺癌的80%-85%。分子分型的出现为晚期NSCLC的治疗开创了全新局面^[2]^。目前针对表皮生长因子受体（epidermal growth factor receptor, *EGFR*）突变、*ALK*融合、*ROS1*融合、MET 14外显子跳跃突变，*BRAF*突变患者的靶向治疗已经为国内外指南推荐的一线标准治疗方案^[3]^。近年来，免疫治疗特别是免疫检查点抑制剂治疗在肺癌领域取得了长足的进展。通过阻断程序性死亡蛋白受体1（programmed cell death protein 1, PD-1）与其配体程序性死亡配体1（programmed cell death ligand 1, PD-L1）相互的作用，解除肿瘤诱导的特异性T细胞活化抑制，达到抗肿瘤效果^[4]^。指南推荐PD-L1表达量高于50%且无驱动基因突变的晚期非鳞NSCLC患者一线可选择帕博利珠单抗单药治疗^[5]^。有研究^[6]^显示，尽管*EGFR*突变患者的PD-L1表达率相较总体人群偏低，但仍有一定比例患者存在驱动基因突变且PD-L1高表达。针对这类患者的临床治疗方案应该如何选择？小样本临床研究^[7]^显示PD-L1高表达的患者一代EGFR酪氨酸激酶抑制剂（EGFR-tyrosine kinase inhibitor, EGFR-TKI）疗效不佳。一项回顾性的*meta*分析^[8]^显示，*EGFR*突变患者二线使用PD-1/PD-L1抑制剂效果不佳。有限的数据似乎揭示出了驱动基因突变且PD-L1高表达患者的临床难治特性，但在真实世界中该类患者临床病理特征及对靶向治疗的反应仍未被完全揭示。本文拟通过对驱动基因阳性且PD-L1高表达的NSCLC患者的病理特征及靶向治疗疗效评估的真实世界研究，对该临床问题进行初步探究。

## 1 材料与方法

### 1.1 患者情况

本研究收集了315例2017年-2019年就诊于首都医科大学北京胸科医院的NSCLC患者。回顾性收集患者的人口学、临床病理学、驱动基因突变状态、PD-L1表达水平、治疗和预后资料。其中仅有1例患者为分子检测数据来源于靶向治疗进展后标本检测结果，其他患者数据均取自一线治疗前标本检测结果。

### 1.2 检测方法

所有患者的肿瘤组织样本均经10%中性福尔马林缓冲液固定后，进行石蜡包埋。驱动基因变异检测：使用FFPE DNA/RNA试剂盒（艾德生物）进行DNA和RNA提取。经多基因检测试剂盒（荧光PCR法）（艾德生物）进行*EGFR*、*ALK*、*ROS1*、*RET*、*KRAS*、*BRAF*、*PIK3CA*、*HER2*和*MET* 9种驱动基因突变检测。所有的检测均在医院病理科，参照产品说明书完成。PD-L1表达水平检测：使用PD-L1 IHC 22C3分析试剂盒，用Dako Autostainer Link 48（ASL48）免疫组织化学染色机。操作方法和数据分析均遵循制造商的说明。按肿瘤肿瘤细胞阳性比例分数（tumor proportion score, TPS） < 1.0%、1.0%-49.0%、≥50.0%分成三组。定义TPS > 1.0%为PD-L1阳性，TPS≥50.0%为PD-L1高表达。

### 1.3 统计学方法

使用SPSS 19.0版软件分析数据。各组间样本率的比较及其与临床病理特征的关系采用*χ*^2^检验。*P* < 0.05被认为具有统计学差异。

## 2 结果

### 2.1 患者临床病理学特征

315例患者的基础信息见表 1，所有的患者都有PD-L1表达水平记录，其中表达量 < 1.0%占58.4%，1.0%-49.0%占29.6%，≥50.0%占11.2%。驱动基因突变情况见图 1，驱动基因总阳性率为62.2%。*EGFR*为主要驱动基因，突变频率为44.8%（141/315），以常见的19外显子缺失和21外显子L858R突变为主。其他驱动基因由突变率高到低依次为*KRAS*突变（7.0%, 22/315）、*ALK*融合（4.1%, 13/315）、HER2 20外显子插入（2.2%, 7/315）、*BRAF*突变（1.3%, 4/315）、*RET*融合（1.3%, 4/315）、*PIK3CA*突变（1.0%, 3/315）、MET 14外显子跳跃突变（0.3%, 1/315）和*ROS1*融合（0.3%, 1/315）。所有驱动基因变异均为单独出现，未测到共突变。PD-L1高表达在驱动基因阳性患者中占比10.7%（21/196），在驱动基因阴性患者中占比为12.6%（15/119），二者无统计学差异（*P*=0.720）。

**1:** 315例NSCLC患者基础病理学特征 Demographic and clinicopathological data of 315 NSCLC patients

Item	*n*	Percentage (%)
Gender		
Male	194	61.6
Female	121	38.4
Age (yr)		
< 65	172	45.4
≥65	143	54.6
Smoking status		
Former	173	54.9
Never	142	45.1
Family history of cancer		
Yes	60	19.0
No	255	81.0
History of chronic diseases		
Yes	36	11.4
No	279	88.6
Clinical stage^*^		
Ⅰ	79	25.1
Ⅱ	21	6.7
Ⅲ	62	19.7
Ⅳ	149	47.3
Pathological type		
Adenocarcinoma	248	78.7
Squamous carcinoma	53	16.8
others	14	4.4
PD-L1 expression level		
< 1.0%	184	57.3
1.0%-49.0%	95	29.6
≥50.0%	36	11.2
NSCLC: non-small cell lung cancer; PD-L1: programmed death ligand 1. ^*^: 4 patients with clinical stage data missing.		

**1:**
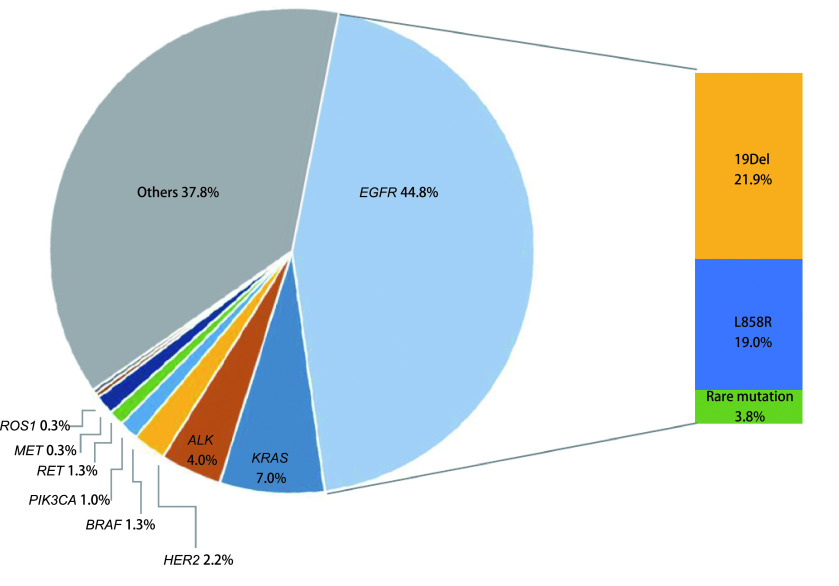
315例NSCLC患者驱动基因突变频谱 Profiling of oncogenic driver mutation in 315 NSCLC patients. The mutation profile of 9 oncogenic driver genes: *EGFR*, *KRAS*, *ALK*, *HER2*, *BRAF*, *RET*, *PIK3CA*, *MET*, and *ROS1* in the enrolled patients. And the variants details of *EGFR*.

### 2.2 驱动基因突变与PD-L1表达水平的关系

各驱动基因阳性患者中PD-L1表达情况，详见图 2。将PD-L1表达水平按 < 1.0%、1.0%-49.0%、≥50.0%分成3组：*EGFR*突变阳性患者中这3组的占比分别为66.7%、25.5%、7.8%；*KRAS*突变阳性的患者这3组的占比分别为36.4%、45.5%、23.1%；*ALK*融合阳性的患者这3组的占比分别为38.5%、38.5%、23.1%，且3组之间存在统计学差异（*P*=0.012）。其他驱动基因阳性患者中，*BRAF*突变阳性和*MET* 14外显子跳跃阳性患者中均有PD-L1表达量超过50.0%的患者，*RET*融合阳性和*ROS1*融合阳性患者则均为PD-L1表达量 < 1.0%，HER2 20外显子插入阳性和*PIK3CA*突变阳性患者PD-L1表达则分布在 < 1.0%组和1.0%-49.0%组。由于携带这些稀有突变患者数太少，PD-L1的表达在这些组中均未有统计学差异。

**2:**
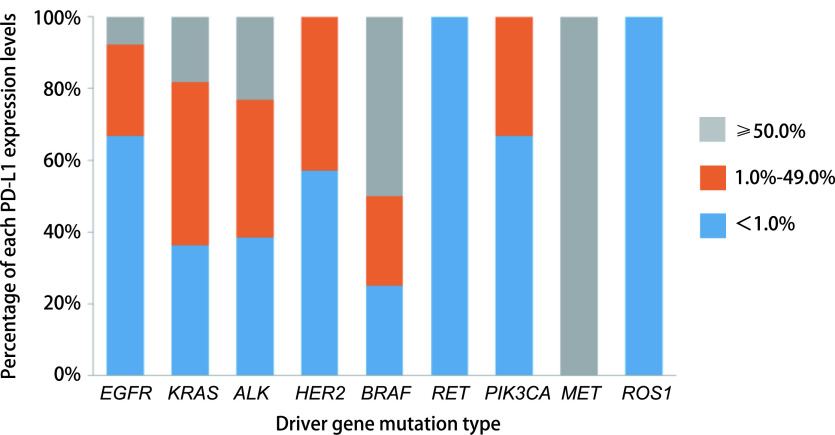
驱动基因突变类型与PD-L1表达水平关系 Relationship between the driver gene mutation type and the PD-L1 expression level.All the 315 enrolled patients were grouped according to their driver gene mutations, and the proportion of each expression level of PD-L1 was counted.

### 2.3 驱动基因阳性患者中PD-L1表达水平与临床病理特征关系

所有入组患者中，PD-L1表达水平在年龄、性别、吸烟史和组织学类型中无统计学差异，而在临床分期中有显著差异（表 2）。PD-L1高表达的患者中，83.5%的患者临床分期为Ⅲ期/Ⅳ期。*EGFR*突变阳性患者中，PD-L1表达水平在年龄、性别和吸烟史中无统计学差异，而在临床分期和组织学类型中有显著差异（表 3）。PD-L1高表达的患者中，90.9%（10/11）的患者临床分期为Ⅳ期；在肺腺癌的占比最高（81.8%, 9/11），而与PD-L1低表达患者相比在肺鳞癌患者中仍有9.1%（1/11）检出率。*KRAS*突变阳性患者中，PD-L1表达水平在年龄、性别、临床分期和组织学类型中均无统计学差异，而在吸烟史中差异显著，PD-L1高表达的患者均有吸烟史（表 4）。*ALK*突变阳性患者中，未发现PD-L1表达水平与病理特征存在相关性（表 5）。

**2:** PD-L1表达水平与临床病理特征关系 Relationship between PD-L1 expression level and clinicopathological characteristics

Item	PD-L1 expression level			*P*
	< 1.0% (*n*=184)	1.0%-49.0% (*n*=95)	≥50.0% (*n*=36)	
Gender				0.956
Male	112 (60.9%)	59 (62.1%)	23 (63.9%)	
Female	72 (39.1%)	36 (37.9%)	13 (36.1%)	
Age (yr)				0.832
< 65	102 (55.4%)	52 (54.7%)	18 (50.0%)	
≥65	82 (44.6%)	43 (45.3%)	18 (50.0%)	
Smoking status				0.138
Former	93 (64.1%)	56 (58.9%)	24 (66.7%)	
Never	91 (49.5%)	39 (41.1%)	12 (33.3%)	
Clinical stage^*^				0.041
Ⅰ	55 (30.4%)	22 (23.4%)	2 (5.6%)	
Ⅱ	10 (5.5%)	7 (7.4%)	4 (11.1%)	
Ⅲ	34 (18.8%)	18 (19.1%)	10 (27.8%)	
Ⅳ	82 (45.3%)	47 (50.0%)	20 (55.6%)	
Pathological type				0.909
Adenocarcinoma	147 (79.9%)	73 (76.8%)	28 (77.8%)	
Squamous carcinoma	28 (15.2%)	18 (18.9%)	7 (19.4%)	
Others	9 (4.9%)	4 (4.2%)	1 (2.8%)	
^*^: 3 patients with Clinical stage data missing in < 1.0% group; 1 patient with Clinical stage data missing in 1.0%-49.0% group.				

**3:** *EGFR*突变阳性患者PD-L1表达水平与临床病理特征关系 Relationship between PD-L1 expression level and clinicopathological characteristics in *EGFR* mutation-positive patients

Item	PD-L1 expression level			*P*
	< 1.0% (*n*=94)	1.0%-49.0% (*n*=36)	≥50.0% (*n*=11)	
Gender				0.454
Male	43 (71.7%)	14 (38.9%)	3 (27.3%)	
Female	51 (54.3%)	22 (61.1%)	8 (72.7%)	
Age (yr)				0.095
< 65	55 (58.5%)	23 (63.9%)	3 (27.3%)	
≥65	39 (41.5%)	13 (36.1%)	8 (72.7%)	
Smoking status				0.959
Former	34 (36.2%)	14 (38.9%)	4 (36.4%)	
Never	60 (63.8%)	22 (61.1%)	7 (63.6%)	
Clinical stage				0.049
vI	35 (37.2%)	14 (38.9%)	1 (9.1%)	
Ⅱ	1 (1.1%)	0	0	
Ⅲ	9 (9.6%)	5 (13.9%)	0	
Ⅳ	49 (52.1%)	17 (47.2%)	10 (90.9%)	
Pathological type				0.022
Adenocarcinoma	92 (97.9%)	36 (100.0%)	9 (81.8%)	
Squamous carcinoma	1 (1.1%)	0	1 (9.1%)	
Others	1 (1.1%)	0	1 (9.1%)	
EGFR: epidermal growth factor receptor.				

**4:** *KRAS*突变阳性患者PD-L1表达水平与临床病理特征关系 Relationship between PD-L1 expression level and clinicopathological characteristics in *KRAS* mutation-positive patients

Item	PD-L1 expression level			*P*
	< 1.0% (*n*=8)	1.0%-49.0% (*n*=10)	≥50.0% (*n*=4)	
Gender				> 0.999
Male	6 (75.0%)	7 (70.0%)	3 (75.0%)	
Female	2 (25.0%)	3 (30.0%)	1 (25.0%)	
Age (yr)				0.740
< 65	5 (62.5%)	5 (50.0%)	3 (75.0%)	
≥65	3 (37.5%)	5 (50.0%)	1 (25.0%)	
Smoking status				0.039
Former	2 (25.0%)	6 (60.0%)	4 (100.0%)	
Never	6 (75.0%)	4 (40.0%)	0	
Clinical stage				0.571
Ⅰ	2 (25.0%)	1 (10.0%)	1 (25.0%)	
Ⅱ	0	4 (40.0%)	1 (25.0%)	
Ⅲ	1 (12.5%)	2 (20.0%)	1 (25.0%)	
Ⅳ	5 (62.5%)	3 (30.0%)	1 (25.0%)	
Pathological type				> 0.999
Adenocarcinoma	8 (100.0%)	10 (100.0%)	4 (100.0%)	
Squamous carcinoma	0	0	0	
others	0	0	0	

**5:** *ALK*融合阳性患者PD-L1表达水平与临床病理特征关系 Relationship between PD-L1 expression level and clinicopathological characteristics in *ALK* fusion-positive patients

Item	PD-L1 expression level			*P*
	< 1.0% (*n*=5)	1.0%-49.0% (*n*=5)	≥50.0% (*n*=3)	
Gender				0.476
Male	1 (20.0%)	3 (60.0%)	2 (66.7%)	
Female	4 (80.0%)	2 (40.0%)	1 (33.3%)	
Age (yr)				0.441
< 65	4 (80.0%)	4 (80.0%)	1 (33.3%)	
≥65	1 (20.0%)	1 (20.0%)	2 (66.7%)	
Smoking status				0.767
Former	1 (20.0%)	3 (60.0%)	1 (33.3%)	
Never	4 (80.0%)	2 (40.0%)	2 (66.7%)	
Clinical stage				0.790
Ⅰ	2 (40.0%)	1 (20.0%)	0	
Ⅱ	0	0	0	
Ⅲ	0	0	1 (33.3%)	
Ⅳ	3 (60.0%)	4 (80.0%)	2 (66.7%)	
Pathological type				> 0.999
Adenocarcinoma	5 (100.0%)	5 (100.0%)	3 (100.0%)	
Squamous carcinoma	0	0	0	
Others	0	0	0	

### 2.4 驱动基因阳性且PD-L1高表达的患者病例分析

病例1：男性，32岁，汉族，吸烟10年，日吸烟量20支。肺穿刺活检病理诊断左肺腺癌，临床分期T1N3M1c，Ⅳ期。肿瘤组织基因检测为*EML4-ALK*融合阳性，PD-L1表达量90%。治疗一线给予克唑替尼单药治疗，1个月后CT评估疗效病情稳定（stable disease, SD），2个月后CT评估为病情进展（progressive disease, PD）。一线无疾病进展时间（progression-free survival, PFS）仅为2.5个月。随后二线给予色瑞替尼治疗，1个月后CT评估肿瘤进展，三线更换为阿来替尼治疗，病情持续进展，1个月后患者死亡（图 3）。患者总生存期（overall survival, OS）为5个月。病例2：女性，67岁，汉族，无吸烟史。肺穿刺活检病理诊断左肺腺癌，临床分期T4N3M1c，Ⅳ期。肿瘤组织基因检测为*EGFR* L858R突变，PD-L1表达量为70%。一线给予埃克替尼治疗，1个月后电子计算机断层扫描（computed tomography, CT）评估为PD。二线换用奥西替尼治疗，仅1个月后患者死亡（图 4）。患者OS为2个月。

**3:**
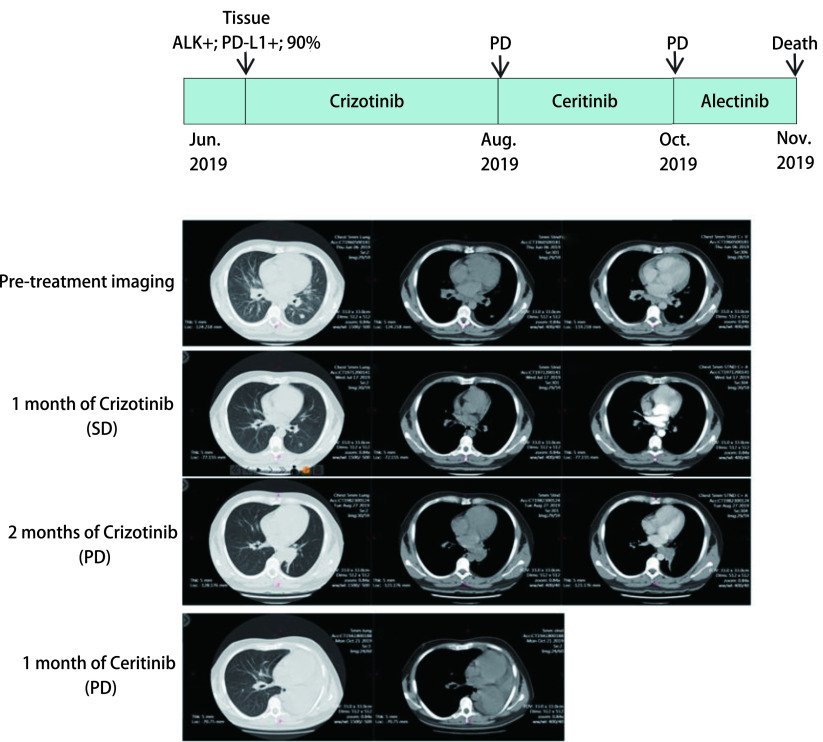
患者1治疗过程以及治疗前后CT诊断图 Timeline of patient evolution and chest computed tomography scans at diagnosis and after treatment in case 1. CT: computed tomography; PD: progressive disease; SD: stable disease.

**4:**
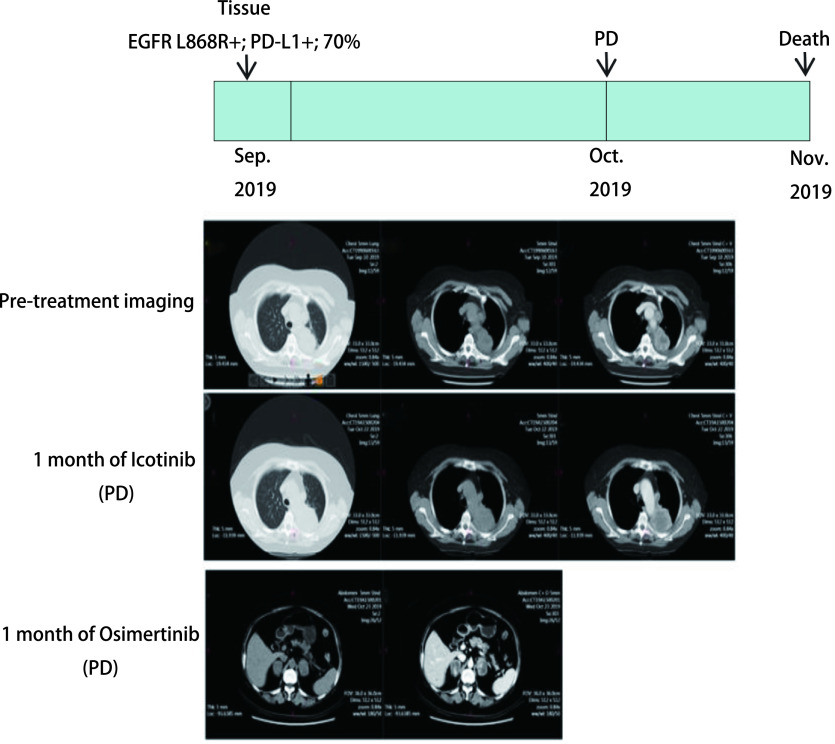
患者2治疗过程以及治疗前后CT诊断图 Timeline of patient evolution and chest computed tomography scans at diagnosis and after treatment in case 2

## 3 讨论

在本研究纳入的315例NSCLC患者中，尽管男性（61.6%）、有吸烟史（54.9%）的患者偏多，但病理分型仍以腺癌（78.7%）为主。9个驱动基因总阳性率为62.2%，其中*EGFR*突变（44.8%, 141/315）、*KRAS*突变（7.0%, 22/315），*ALK*融合突变（4.1%, 13/315）为主要的三种驱动基因，与已报道的数据^[9, 10]^相符。所有患者中，PD-L1表达水平整体偏低，高表达患者占11.2%，表达量在1%-49%的病例占29.6%，该数据也与已报道的数据相符^[11]^。2020年发表于*Ann Oncol*上关于非鳞NSCLC的PD-L1表达水平与临床病理特征的大样本数据^[12]^显示，PD-L1高表达与烟草暴露量增加，临床晚期和较高的肿瘤突变负荷相关。本研究未发现PD-L1表达水平与吸烟史的关系，但验证了其与临床分期的关系。本研究数据显示，PD-L1高表达与PD-L1低表达和无表达的患者相比，更多的富集在Ⅲ期/Ⅳ期患者中，三组中Ⅲ期患者的比例分别为27.8%、19.1%和18.8%，Ⅳ期患者比例分别为55.6%、50.0%和45.3%。目前多项体内外研究报道了NSCLC驱动基因突变与PD-L1表达的相关性。体外研究^[13]^显示EGFR信号通路激活后可通过磷酸化ERK1/2/c-JUN，或激活JAK-STAT3、PI3K/AKT信号通路来上调PD-L1的表达。全球多中心真实世界EXPRESS研究，纳入局部晚期或转移性NSCLC患者，其中*EGFR*突变阳性且PD-L1表达水平≥50%患者占比为13%（60/448），≥1%患者比例为44%（197/448）^[14]^。本研究中，*EGFR*突变阳性患者中，PD-L1高表达的比例为7.8%，PD-L1阳性比例为33.3%，与EXPRESS研究数据相比比例偏低。这可能与本研究纳入了31.8%的Ⅰ期-Ⅱ期患者相关。进一步分析患者的病理特征发现*EGFR*突变且PD-L1高表达患者中90.9%（10/11）临床分期为Ⅳ期，且相比于低表达患者肺腺癌的占比更高（9.1%, 1/11）。研究^[13]^显示*ALK*融合可与PD-L1高表达伴发，其分子机理为ALK信号通路激活后通过磷酸化ERK1/2/c-JUN来上调PD-L1的表达。EXPRESS研究^[14]^中，*ALK*融合阳性且PD-L1表达水平≥50%患者占比为20%（15/74），≥1%患者比例为65%（48/74）。本研究中*ALK*融合阳性患者中，PD-L1高表达的比例为23.1%，PD-L1阳性比例为61.5%。细化分析患者病理特征，未发现与PD-L1表达水平显著相关的特征，这可能与研究病例数较少相关。研究^[15]^显示，*KRAS*突变通过激活ERK信号通路上调PD-L1表达水平。与*KRAS*野生患者相比，*KRAS*突变患者更易为PD-L1表达阳性（51% *vs* 36%; OR=1.69, 95%CI: 1.01-2.84, *P*=0.045）^[16]^。本研究中*KRAS*突变阳性患者中，PD-L1高表达患者比例为23.1%，PD-L1阳性比例为68.6%，且所有PD-L1高表达的患者均有吸烟史。在肠癌和甲状腺癌相关研究^[17, 18]^中均显示*BRAF*突变与PD-L1高表达相伴发生。一项关于951例NSCLC患者的数据^[19]^显示，*MET* 14外显子跳跃突变患者中PD-L1高表达的比例为69.2%。本研究中共4例*BRAF*突变和1例*MET* 14外显子跳跃突变患者，其PD-L1高表达的比例分别为50%和100%。尽管病例数有限，但本研究数据仍可反映出驱动基因改变与PD-L1表达的相关性。本研究发现携带如下驱动基因改变患者PD-L1表达量偏低，如HER2 20外显子插入、*PIK3CA*突变、*RET*融合、*ROS1*融合。这些发现尚有待大样本量数据的验证。

本研究详细追踪了2例初诊患者诊断和治疗过程。2例患者均为腺癌，携带敏感突变且PD-L1高表达。患者接受靶向治疗后快速进展，即便更换成二代或三代EGFR-TKI仍无法有效控制肿瘤增大，患者OS均未超过半年。2例患者诊疗全程中，没有合适的机会取材做更大panel的基因测序以探究基因组层面的变异。仅有的数据提示，携带敏感突变且PD-L1高表达的患者可能有更差的预后，且靶向治疗获益有限。在一项针对101例*EGFR*敏感突变阳性的NSCLC患者研究中^[7]^，显示PD-L1的高表达显著降低了EGFR-TKI药物治疗客观缓解率（objective response rate, ORR）（35.7%），缩短了PFS（3.8个月）。此外，在原发耐药的患者中观察到PD-L1阳性表达高于EGFR-TKI的获得性耐药患者（66.7% *vs* 30.2%, *P*=0.009）。针对这类患者应当给予何种治疗方案，已成为临床亟待解决的问题。尽管驱动基因突变阳性及PD-L1高表达分别为靶向药物和免疫药物的适应症，但两药的联合似乎并不能达到很好的效果。一项Ⅰ期研究^[20]^评估了纳武单抗联合厄洛替尼治疗*EGFR*突变晚期NSCLC的疗效。所有患者接受每2周纳武单抗，每天150 mg的厄洛替尼治疗。20例EGFR-TKI经治患者的ORR为15%，中位PFS和中位OS分别为5.1个月和18.7个月。5例患者出现3级治疗相关不良反应，其中2例因不良反应停药。一项Ⅰ期/Ⅱ期研究^[21]^探索了帕博利珠单抗联合厄洛替尼或吉非替尼一线治疗携带*EGFR*敏感突变的晚期NSCLC患者。所有患者每3周接受帕博利珠单抗2 mg/kg治疗，厄洛替尼联合组每天口服150 mg厄洛替尼；在吉非替尼联合组每天口服250 mg吉非替尼。两组有效率分别为41.7%（12例患者厄洛替尼联合组）和14.3%（7例患者吉非替尼联合组）。厄洛替尼治疗组的耐受性尚可，但吉非替尼治疗组中有5例（71.4%）患者发生3级/4级转氨酶升高，且4名患者因此停药。Ⅰ期TATTON研究^[22]^纳入既往EGFR-TKI治疗进展的*EGFR*突变的NSCLC患者。剂量扩增队列中每天口服奥美替尼80 mg，或每2周静脉注射3 mg/kg-10 mg/kg度伐利尤单抗。研究结果显示有38%（13/34）的患者在奥希替尼联合度伐利尤单抗治疗时出现间质性肺炎，其中5例患者为3级/4级。而奥希替尼或度伐利尤单抗单药治疗时间质性肺炎发生率仅分别为2.9%和2.0%。由于间质性肺炎发生率过高，该研究及Ⅲ期CAURAL研究^[23]^也因此终止。因此，免疫联合EGFR-TKI未明显提高疗效，反而显著增加了毒副作用，且不同药物组合之间的毒性反应也存在差异。免疫联合靶向治疗是否在驱动基因阳性且PD-L1高表达患者中有效还有待更多研究探究，这之前可能还需摸索出合理的用药顺序和时间间隔。已有研究显示，免疫抑制剂治疗单药治疗似乎有获益趋势。Ⅱ期ATLANTIC研究^[24, 25]^显示，度伐利尤单抗作为三线及三线以上方案治疗*EGFR*/*ALK*突变的NSCLC患者，PD-L1表达≥25%组相较于低表达组应答率更高（43% *vs* 22%），且在中位OS（13.3个月*vs* 9个月）、12个月OS率（53.3% *vs* 40.4%）、24个月OS率（40.7% *vs* 14.7%）具有更优表现。亚组分析显示*EGFR*突变阳性比*ALK*阳性患者的中位OS更优，分别为16.1个月和6.3个月。BRICH研究^[26]^探究了阿特珠单抗作为一线及后线治疗PD-L1表达超过5%的NSCLC患者疗效。纳入患者中，8%*EGFR*突变阳性，28%*KRAS*突变阳性，2%*ALK*融合阳性，46%PD-L1高表达。其中PD-L1高表达亚组（TC≥25%或IC≥10%）的ORR为26%-31%；大多数病例仍在进行中。尽管研究公布无论*EGFR*或*KRAS*突变状态如何，均会发生反应，但未给出驱动基因阳性且PD-L1高表达患者与无基因变异的PD-L1高表达患者对比疗效。然而，另外有部分研究显示，*EGFR*突变可能还是免疫治疗发生超进展的相关风险因子。程颖教授团队^[27]^回顾性分析了74例组织学确诊的IIIb期或Ⅳ期NSCLC患者，二线或后线接受免疫单药治疗疗效情况，其中*EGFR*/*ALK*突变患者为经靶向治疗后进展。中位随访时间为14.1个月(95%CI：1.7个月-39.3个月)，64例（86.5%）患者有影像学确诊的疾病进展，其中25例患者快速进展，39例患者为非快速进展。进一步分析快速进展相关的影响因素发现，快速进展与美国东部肿瘤协作组（Eastern Cooperative Oncology Group, ECOG）2分、*EGFR*/*ALK*突变、转移灶超过3个和中性粒细胞/淋巴细胞比率≥3显著相关。免疫联合化疗似乎也给这类患者带来希望。CT18研究^[28]^是免疫联合化疗二线挑战EGFR-TKI治疗失败的NSCLC患者的单臂研究。该研究入组40例*EGFR*突变患者为先前接受EGFR-TKI治疗失败同时未伴有T790M突变或接受奥希替尼治疗失败。接受特瑞普利单抗联合卡铂和培美曲塞4个-6个周期，后续接受特瑞普利单抗联合培美曲塞维持治疗，直至疾病进展或不能耐受。研究结果表明ORR为50%（95%CI: 33.8%-66.2%），中位PFS为7.0个月（95%CI：4.8个月-10.3个月）。其中PD-L1高表达（≥50%）且*EGFR*突变的患者的ORR（29.4%）及PFS（5.3月）有获益趋势。IMpower150研究^[29]^中在接受ABCP治疗组（阿特珠单抗+贝伐珠单抗+卡铂+紫杉醇）中9%患者*EGFR*突变阳性，3%患者*ALK*融合阳性，19%患者PD-L1高表达；BCP治疗组（贝伐珠单抗+卡铂+紫杉醇）11%患者*EGFR*突变阳性，5%患者*ALK*融合阳性，18%患者PD-L1高表达。按驱动基因变异分层分析，*EGFR*阳性患者中，ABCP治疗组比BCP治疗组，有效率更高（71% *vs* 42%），中位缓解持续时间更长（11.1个月*vs* 4.7个月），中位PFS（10.2个月*vs* 6.9个月）和OS均有优势（未达到*vs* 18.7个月）。但是*ALK*融合阳性的患者ABCP组较BCP治疗组在PFS没有统计学差异（8.3个月*vs* 5.9个月）。若按PD-L1表达水平进行亚组分析，PD-L1高表达的患者，ABCP组较BCP治疗组在OS上差异显著（25.2个月*vs* 15.0个月）。研究未公布驱动基因阳性且PD-L1高表达患者的治疗对比数据。因此，对于携带敏感突变且PD-L1高表达的患者，能从何种治疗方案中获益，还有待前瞻性临床研究的探究。

综上所述，本研究通过对真实世界数据的回顾性分析，发现各驱动基因突变的NSCLC患者PD-L1高表达的比例各不相同，且高表达患者临床病理特征也较低表达或阴性患者有较大差异。携带*EGFR*突变、*ALK*融合的患者且PD-L1高表达时，靶向治疗获益有限，预后可能更差。这些发现提示在临床实践中该类患者应给予重点识别和特殊治疗，也亟需更多的大型前瞻性临床研究。
